# 

**Published:** 1873-01

**Authors:** 


**CONTENTS:**

ORIGINAL COMMUNICATIONS:

ARTICLE I. Clinical Lectures on Diseases of the Ear. By E. L. Holmes, M.D., Chicago.......... 1

ART. II. Lecture 3d—Neuralgia (continued). By W. Hay, M.D., Chicago. 4

ART. III. Diphtheritic Ophthalmia Treated with Carbolic Acid and Iodine. By F.C. Hotz, M.D., Chicago 16

ART. IV. The Present Status of the Pessary in Gynecological Practice. By Philip Adolphus, M.D., Chicago. 18

SELECTIONS.

The Effect of Cold upon the System.. 22

Thermometry in Health and Disease. 30

On the Physical Education and Training of Children.......... 38

Dr. Ricord on Syphilis,.......... 46

Dropsy of the Amnios—Operation, Recovery.......... 54

Small-Pox Statistics.......... 56, 57

EDITORS' BOOK TABLE.......... 58

EDITORIAL.......... 60

